# Parental COVID-19 Vaccine Hesitancy for Children in Romania: National Survey

**DOI:** 10.3390/vaccines10040547

**Published:** 2022-04-01

**Authors:** Flavius Cristian Mărcău, Cătălin Peptan, Ramona Mihaela Nedelcuță, Vlad Dumitru Băleanu, Anca Roxana Băleanu, Bogdan Niculescu

**Affiliations:** 1 Faculty of Educational Sciences, Law and Public Administration, “Constantin Brâncuși” University of Târgu Jiu, 210185 Târgu Jiu, Romania; flavius.marcau@utgjiu.ro (F.C.M.); catalin.peptan@utgjiu.ro (C.P.); 2 Department of Pediatrics, University of Medicine and Pharmacy of Craiova, 200349 Craiova, Romania; 3 Faculty of Medical and Behavioural Sciences, “Constantin Brâncuși” University of Târgu Jiu, 210185 Târgu Jiu, Romania; vlad.baleanu@utgjiu.ro (V.D.B.); bogdan.niculescu@utgjiu.ro (B.N.); 4 Department of Anesthesiology and Intensive Care, University Emergency Hospital Bucharest, 050098 Bucharest, Romania; ancaroxanapascal@gmail.com

**Keywords:** anti-COVID-19 vaccine, children vaccination, hesitation, Romania

## Abstract

Purpose: Once vaccination against COVID-19 was also possible for children over 12 years of age, parents/legal guardians had to give their consent for their vaccination. It is a crucial moment, given the large number of infected people in Romania and the fact that these children are a source of transmission of the virus in the community. The refusal or hesitation of the parents/legal guardians, regarding the agreement for the vaccination of the children, determined us to focus on this subject, wishing, based on the questioning of as many parents as possible, to extract the reasons underlying these decisions. Methods: This study is designed to observe the attitudes of parents/legal guardians regarding the refusal, hesitation, or acceptance of vaccination of children. The persons targeted to answer the questionnaire had to meet three conditions: to be at least 21 years old, to have a stable residence in Romania, and to be parents/legal guardians of at least one child under 18 years of age. The questionnaire was applied online to a number of 581 parents/legal guardians, being structured to obtain socio-demographic data and other categories of data that allow us to analyze their views on vaccinating children. Results: Sociological data resulting from the application of the questionnaire on 581 parents/legal guardians show that 183 (31.5%) adults and 140 (24.1%) children got infected with COVID-19. The total number of respondents shows that only 411 (70.7%) adults and 185 (31.8%) children are vaccinated. Conclusions: From the analysis of the data obtained through the questionnaire, following the application of the Kendall and Spearman statistical analysis tests, it is found that there is a strong link between participants’ trust/distrust in “fake news” information and their decision to vaccinate their children.

## 1. Introduction

In March 2020, the World Health Organization (WHO) declared COVID-19 viral disease a pandemic. It was caused by the newly identified coronavirus SARS-CoV-2 (Severe Acute Respiratory Syndrome Coronavirus 2), the name given by the WHO to the new coronavirus strain, discovered in 2019 in the Chinese city of Wuhan. The pandemic is in a continuous, unpredictable dynamic, due to the new mutations of the coronavirus that appear periodically, becoming dominant in a very short time.

By the time of this study, February 2022, there were 5,830,272 deaths worldwide [1], while in Romania the death [2] toll reached 61.520 to 2.559.348 reported cases of infection [3].

Experts estimate that the COVID-19 pandemic is far from being over, and its economic and social consequences are disastrous. The fifth wave is in full swing, making many victims around the world.

Romania, during the fourth wave caused by the Delta variant, went through a very serious pandemic period [4], with records in terms of the number of infected, hospitalized in intensive care, and deceased people [5]. The fifth wave brought new records for the number of infected people reported daily, but the number of deaths remains low. The measures implemented by the government to limit the spread of the virus have not yielded the expected results, thus an effective vaccination campaign remains the key variable in the fight against the virus and return to normality. The public rhetoric related to the establishment of the green certificate on the Romanian territory, as well as in many European countries, generated an additional state of tension among the citizens, without, however, any substantial increase in the vaccination process.

The effects of the pandemic seriously affected the category of young people who are the subject of this study. The shifting of educational process in the online environment was likely to affect its quality, including the right of children to education. Medically, beyond the adverse consequences in terms of psychological comfort of children and their families, the COVID-19 pandemic has affected people with various comorbidities, due to the additional risks they were subject to in case of infection with the new coronavirus [6]. This, in the case of infecting young people, raises serious problems for those who have comorbidities and are likely to develop severe forms [7,8]. It should be noted that, when children and adolescents come into contact with the virus and become infected, they often remain asymptomatic or develop mild symptoms of the disease that remit within a few days, and hospitalization rates, intensive care, or deaths are extremely low.

We believe that vaccinating young people is beneficial both from a medical point of view, to prevent a potential infection, and from a psychological point of view, as it offers additional guarantees regarding the development of a normal social life. Vaccination of children is an essential policy to combat the pandemic, due to the fact that they can be serious vectors of spreading the disease [9,10,11] within their families [12]. On the other hand, they may be “major factors of active epidemic waves (in terms of their share in the number of documented cases), especially with the Delta variant” [13].

Our study focuses on determining the degree of acceptance, refusal, or hesitation of parents/legal guardians regarding the anti-COVID-19 vaccination of children under 18 years of age.

The aim of the research is to obtain essential data to answer the questions set out in our study, as shown in Table 1. In this way we will be able to know if there are links between these data and the reasons for accepting/rejecting COVID-19 vaccines.

## 2. Research Materials and Methods

### 2.1. Participants

The study was conducted following the application of a questionnaire in the online environment, due to the impossibility of applying it in the “face to face” variant, as national authorities of restrictions aimed at limiting the spread of coronavirus in communities. The questionnaire was applied in Romania, between 4–11 December 2021 (at the end of the fourth wave of the pandemic), to persons who are parents/legal guardians and have children under the age of 18. Any willing person could participate in filling in the questionnaire. However, the minimum conditions required for a person to be included in the target group were the following: (1) minimum age of 21 years old; (2) to be the parents or the legal guardians and the children must be under 18 years of age, so that the parents are the ones who decide on vaccination/non-vaccination; (3) permanent residence in Romania as the study was carried out in Romania.

The participation was voluntary, with no compelling elements. All respondents received information, included from the first page of the questionnaire, about the authors, their affiliation, purpose, and source of research funding.

### 2.2. Procedure

Volunteers received a questionnaire created on the Google Forms platform. No identification data of the respondents were requested, and it was allowed to distribute the form, based on the link, on the printed from. Completion was possible only for people who ticked “Yes” to the question regarding the status of parent or legal guardian of a child under 18 years of age.

The questions in the questionnaire were in Romanian, constructed in order to obtain information (as shown in Table 1) about parents/legal guardians and their children. This information allowed us to answer the questions set out in the introduction section. Additionally, the “fake news” was built based on the false information found on social networks.

The distribution of the questionnaire was performed on social platforms, aiming to cover, from a geographical point of view, at least 70% of Romania’s counties.

### 2.3. Measurements

Collecting the data allowed us to establish the behavioral attitude of parents/legal guardians regarding the acceptance, non-acceptance, or hesitation of vaccinating their own children. The aim was to obtain: socio-demographic data (age, place of residence, educational background, county of residence); data on the respondents’ opinions on the acceptance/non-acceptance of vaccines included in the mandatory vaccination schedule in Romania (hepatitis B vaccine, hexavalent vaccine, tuberculosis vaccine, etc.) and optional vaccines for children (like rotavirus vaccine, hepatitis A and B, meningococcal vaccine etc.); data on the confidence in COVID-19 vaccines available to minors; data regarding the acceptance/non-acceptance of the administration of the child/children vaccine; motivation for accepting/not accepting the administration of the vaccine.

We also aimed to establish the participants’ behavior regarding the “fake news” statements related to the COVID-19 vaccination process [14].

### 2.4. Statistical Analysis of Data

The analysis and processing of the extracted data, after questionnaire filling, was performed using the Excel program, part of Microsoft Office Professional Plus 2019, and IBM SPSS Statistics 26. These were installed on a computer with an operation system Microsoft Windows 11 Professional. The data collected through the questionnaire was automatically centralized in an excel file and followed by the processes of visualization, extraction, and statistical analysis.

The variables used for the analysis of data regarding the respondents are the following: (1) age group; (2) acceptance/non-acceptance of mandatory and optional vaccines; (3) acceptance of COVID-19 vaccination of parents/legal guardians; (4) trust/mistrust of parents/legal guardians in “fake news”. Following the statistical analysis, we proceeded to compare the results to observe the behavior of parents/legal guardians according to the four variables mentioned above, presenting the data where we observed a significant difference.

The processing and extraction of data from the answers given to the open-ended question was performed by hand, as the analyses extracted using AI-based programs were not conclusive enough. We reviewed all the answers provided, together with the centralization of the reasons provided by the parents, in order to determine the final percentages for our study.

The answers in the last part of the questionnaire helped us to understand the level of trust given to the information found in the “fake news” [14].

Various statistical analysis tests were applied to determine the dependence between the selected variables. Thus, we applied statistical tests that helped us to understand whether there is a link between vaccination of children with vaccines included in the national vaccination schedule, optional vaccines and parents’ decision to vaccinate/not vaccinate their children against COVID-19; participants who rated 1–2 (disagree) and 4–5 (agree) on the “fake news” statements were selected and correlated with their decisions to vaccinate/not vaccinate their children against COVID-19 to understand if there is a relationship between these variables.

## 3. Results

The present study includes the analysis of a number of 581 valid answers, provided through the applied questionnaire. The socio-demographic data of the participants are presented in Table 2.

### 3.1. Infections with COVID-19 Disease

From the extracted data, it results that a number of 323 people, representing 55.5% of the total respondents, had at least one positive test that highlights the infection with COVID-19 disease. Of these, 183 (31.4%) were adults and 140 (24%) were children. The graphical representation of the number/percentage of infected people with COVID-19, by age groups and categories (adults, respectively, children), is presented in Table 3.

### 3.2. Confidence in the Medical System

The statistical processing of the collected data shows that the respondents’ trust in the Romanian medical system (47.6%) is significantly low compared to the trust given to doctors (87%). The result is worrying and, we believe, this is not only due to the latest incidents in Romanian hospitals, but to the problems faced by the medical health system in recent years, such as: outdated structure of the system [15], very high hospitalization rate, underfunding highlighted [16] by the fact that the level of health expenditure is the lowest in the EU [17], the very high deficit of specialists, and the lack of essential medicines corroborated with the need for their purchase by patients.

### 3.3. Analysis on Vaccination of Participants

The analysis of the data collected on the vaccination of adults and their own children with the vaccines included in the mandatory national scheme reveals a very low rejection rate (Table 4).

The comparative analysis of the confidence rate between the vaccines included in the mandatory national scheme in Romania and the optional vaccines is presented in Table 5. We observe that the degree of acceptability of compulsory vaccines and optional vaccines, not yet included in the national vaccination scheme, is close.

The analysis of the data collected regarding the situation of anti-COVID-19 vaccination of adults and their children is presented in Table 6.

There is a higher vaccination rate for adults than for their own children. However, the data collected show a relatively low degree of parental/legal guardianship agreement regarding the vaccination of children when they reach the mandatory minimum age or when vaccines will be available for their age category (Table 7).

The level of readiness of people vaccinated with one of the anti-COVID-19 vaccines, to vaccinate their children against the SARS-CoV2 virus, is highlighted in Table 8. We observe that for the age categories older than 40 years of the parents/legal guardians, the level of availability is clearly higher than the one registered for the younger age categories.

There is a very low level of readiness of parents/legal guardians who chose not to vaccinate against COVID-19 to agree that their own children will receive such a vaccine (Table 9).

To the open question “What is the reason why you did/did not vaccinate your child?”, the centralization of the answers reveals that the people who chose to vaccinate their children, or who will vaccinate them when possible, have the following arguments: the need to be healthy (33.6%), avoiding complications caused by COVID-19 (28.7%), avoiding government restrictions (12.2%) and discrimination (8.6%), other reasons (16.7%). Those who have not vaccinated their children gave the following reasons: side effects of undeclared COVID-19 vaccines (33,7%) vaccines are experimental (19.6%), lack of liability of manufacturers, doctors, or government in the case of serious adverse effects (12.5%), dangerous and unstable substances in vaccines (9.9%), children do not develop severe forms of the disease (8.1%), other reasons (16%).

### 3.4. Analysis of Participants’ Confidence in “Fake News” Information

In the second section of the questionnaire, we considered the behavioral analysis of the participants regarding the trust they have in the “fake news” type of. We used statements most often found in the public space when conspiracy theories are discussed. The obtained results are presented in Table 10 [14].

## 4. Discussion

Global studies addressing the issue of vaccination in children [18,19,20,21] show an increase in situations of rejection or hesitation to administer vaccines for various reasons, without being able to identify a valid universal pattern [22] that could lead to a review of such behavior [23], thus inherently ensuring a high degree of immunization against new strains of coronavirus. In this study, we wanted to determine the rejection rates of COVID-19 [24] vaccines and the reasons behind them [25].

In Romania, during the fourth wave, from October to November 2021, a decision for students in pre-academic education was taken to have a two-week vacation in order to limit the spread of the virus. Such measures were also adopted by other states during the pandemic [26]. Children can be a very serious vector of spreading the disease in their own families and in the communities they belong to. Such preventive measures showed a decrease in the number of cases.

From the participants in the study, we observed that 183 (31.5%) adults and 140 (24.1%) children got infected with COVID-19, but regarding vaccination against COVID-19, out of the total respondents only 411 (70.7%) adults and 185 (31.8%) children were vaccinated.

**Figure 1:**
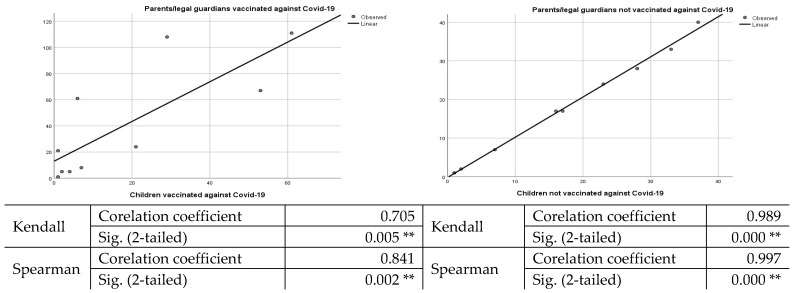
Correlation between parents/legal guardians and children vaccinated/not vaccinated against COVID-19. **. Correlation is significant at the 0.01 level (2-tailed).

The age groups most reluctant to vaccinate their children are 26–30, 31–35, 36–40, and 41–45 years old (Table 6). A comparative analysis regarding the agreement of the parents/legal guardians concerning the vaccination of the children, in the case of the vaccinated parents/legal guardians vs. unvaccinated parents/guardians, pointed out that the acceptance rate of the latter is extremely low. We should mention that, in the case of vaccinated parents/legal guardians, 43.8% of the children were also vaccinated. However, after applying the Kendall and Spearman statistical tests, we observed that there was no significant correlation between parental vaccination against COVID-19 and vaccination of children. However, in the case of parents not vaccinated against COVID-19 and unvaccinated children, the correlation is strong and significant, as shown in Figure 1.

For children who have not reached the minimum age to be vaccinated or the vaccine is not yet available for their age, 77.8% of vaccinated parents/guardians responded that they will vaccinate their children when possible. In the case of unvaccinated parents/guardians, only 3.5% stated that they would vaccinate their children against COVID-19 when possible.

We observe a vaccine reluctance only in the case of anti-COVID-19 vaccines, because when we discuss vaccination (Table 4) and acceptability (Table 5) of vaccines included in the national mandatory scheme or optional vaccines, the percentages are considerably increased, and similar statistics are also found in other states [27,28,29]. In this situation, we have 98.4% adults and 97.2% children vaccinated with the vaccines included in the mandatory national vaccination scheme. In the case of optional vaccines, 51.1% of children are vaccinated with one of them, the percentage being 19.2% higher than in the case of vaccination with one of the COVID-19 vaccines.

**Figure 2:**
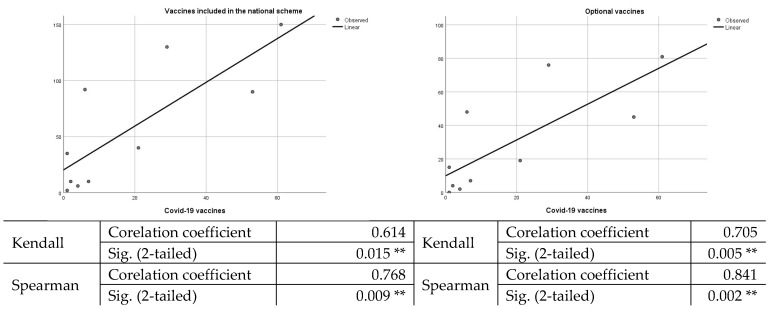
Graphic representation of the correlation between the acceptance of parents/legal guardians of vaccines included in the national vaccination schedule, optional vaccines, and the COVID-19 vaccine. **. Correlation is significant at the 0.01 level (2-tailed).

Statistical analysis carried out to determine a potential link between vaccination of children with vaccines included in the mandatory national schedule, optional vaccines, and acceptance of COVID-19 vaccines shows that this hypothesis is rejected, as there is no strong correlation between variables, as shown in Figure 2.

Comparative analyses among vaccines included in the national scheme, optional vaccines (like rotavirus vaccine, hepatitis A and B, meningococcal vaccine etc.), and anti-COVID-19 vaccines, show a fear about the last category of vaccines. This occurs due to the element of novelty and/or distrust developed against the background of the lack of a minimum education in the medical field. In addition to these arguments, the fake information present in the online environment (sites and social platforms) induces a state of insecurity for people who do not distinguish between “fake news” and scientific data, leading to various situations in which anti-vaccine arguments come from such sources and have no scientific basis.

To the open question “What is the reason why you did/did not vaccinate your child?”, the centralization of the answers reveals that the people who chose to vaccinate their children, or who will vaccinate them when possible, have motivated, as a first argument, the need to be healthy and to avoid complications in the event of a potential infection. The confidence of those who chose the vaccine is validated on the data obtained from doctors, as they declare, also based on the confidence in the vaccines on the market, other than those against COVID-19. The second argument is mobility limitation due to restrictions imposed by the authorities and not necessarily out of a desire to be protected from infection. The third argument is about returning to a normal life, as it was before the pandemic began.

On the contrary, those who have refused to vaccinate their children consider, as a first argument, that vaccines available for children are experimental and no one, pharmaceutical manufacturers or doctors, assumes any potentially dangerous side effects. In addition, parents/guardians, as a second argument, also claim that a very short time has elapsed since the appearance of the vaccines, so all the possible side effects in the medium and long term are not known and there is no research to confirm or negate these potential side effects.

Regarding the section dedicated to “fake news” statements, we notice a high percentage of respondents show confidence in them, so that 22.5% believe that there is a global society wanting to control the world and 20.3% believe that this society wants to reduce the world population. A percentage of 12.3% believe that doctors receive money to inoculate the COVID-19 vaccine to reduce the population.

When questioning “fake news” statements about COVID-19 vaccines, 17.3% of respondents believe that vaccines based on mRNA technology produce dangerous genetic changes, 14.6% believe that vaccination against COVID-19 aims to reduce the number of elderly people, 7.9% believe that the vaccine causes infertility and 7.7% believe that vaccinated people will die in the coming years due to it. Regarding the statement concerning the implantation of a chip in the body by means of the vaccine, 3.6% of the respondents share such an opinion. People who believe such fake news are likely to refuse vaccination, backing up their decisions with false information obtained from such sources.

**Figure 3:**
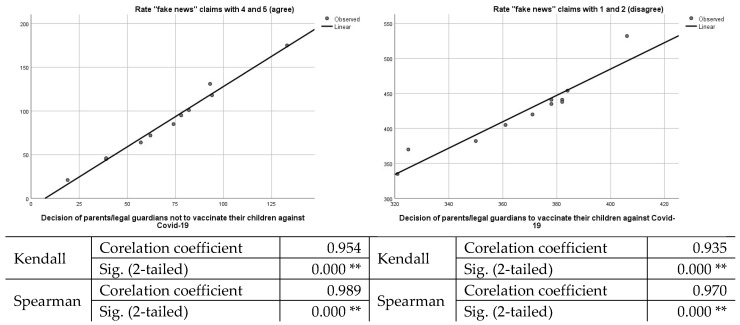
Graphic representation of the correlation between individuals who trust “fake news” (grading 4 and 5) and their decision to vaccinate their children. **. Correlation is significant at the 0.01 level (2-tailed).

Thus, in order to determine the connection between the trust in ”fake news” and the decision not to vaccinate their children, we chose to perform a statistical analysis, having as variables the individuals who graded with 4–5 (agree), 1–2 (disagree), and their decision to vaccinate/not vaccinate their children against COVID-19. The results are presented in Figure 3.

Based on the statistical tests conducted, we consider the hypothesis valid that the level of confidence in “fake news” claims determines the level of vaccination among children. The more parents trust official data and reject “fake news” claims, the greater the acceptance of the COVID-19 vaccine for children.

## 5. Research Limitations

This study has many good aspects, but also some limitations, in the context in which it is among the first studies in Romania to address the issue of acceptance, hesitation, or rejection of COVID-19 vaccines by parents when it comes to vaccinating their own children.

The first important limitation that could reveal a much higher degree of rejection of vaccination is the low proportion of respondents with secondary education compared to the rest of the respondents, in the context in which this layer has a much higher hesitation or rejection of the vaccine.

The second limitation concerns the lack of a question by which we could observe the behavior of parents/legal guardians whose children have comorbidities. Such an approach would have allowed us to observe the degree of acceptability of COVID-19 vaccines in such cases.

## 6. Conclusions

In Romania, the decision to vaccinate minor children against COVID-19 raises a serious problem among parents/legal guardians, divided into two categories: pros and cons of the vaccine. However, vaccination of minors is an important advantage when the vector of the spread of the COVID-19 pandemic by young people is under discussion. In fact, minors, in the case of a potential infection, develop quite mild forms of the disease or remain asymptomatic. Even those with comorbidities rarely develop severe forms or die.

From the analysis of the data obtained through the questionnaire, following the application of the Kendall and Spearman statistical analysis tests, it is found that there is a strong link between participants’ trust/distrust in “fake news” information and their decision to vaccinate their children. Most likely, the lack of minimal medical knowledge among participants and their reliance on malicious or scientifically unsubstantiated information, to the detriment of official information presented by doctors, leads to decisions to reject vaccination.

In addition, we believe that social platforms and some news sites are easy ways of disseminating “fake news” information. More and more authorized voices among medical scientists claim that these platforms are responsible, up to a large extent, for the high degree of rejection of COVID-19 vaccines [14,30,31,32,33,34].

## Figures and Tables

**Table 1:** Required dates and research questions.

Required Dates	Questions
Vaccination of children with vaccines included in the national vaccination schedule in Romania	Does the high uptake of vaccination with vaccines included in Romania’s national schedule and optional vaccines increase the acceptability of vaccination with a COVID-19 vaccine?
Vaccination of children with optional vaccines recommended for children (hepatitis B vaccine, hexavalent vaccine, tuberculosis vaccine, etc.)	
COVID-19 vaccination of parents	Does vaccination/non-vaccination of parents/legal guardians with an COVID-19 vaccine influence the vaccination decision of their children?
COVID-19 vaccination of children	
Parents’ trust in “fake news” claims	Does a high reliance on “fake news” lead to the rejection/refusal to vaccinate children with a COVID-19 vaccine?

**Table 2:** Representation of socio-demographic data of participants.

Age	Sex			Environment of Residence		Educational Level				
	Female	Male	Do Not Answear	Urban	Rural	Secondary Education	High School	Faculty	Masters	Phd
21–25	9	3	-	10	2	1	5	3	3	-
26–30	33	5	-	26	12	1	6	12	16	3
31–35	80	13	1	72	22	-	26	34	30	4
36–40	119	16	1	101	35	2	14	66	46	8
41–45	133	16	2	125	26	3	19	85	39	5
46–50	76	15	-	76	15	2	13	41	29	6
51–55	33	7	1	32	9	-	9	20	9	3
56–60	8	2	-	8	2	-	3	5	1	1
61–65	2	-	-	2		-	-	1	1	-
66+	5	1	-	5	1	-	4	2	-	-

**Table 3:** Infections with COVID-19 disease of respondents and their children.

Age Range for Parents/Legal Guardians	Parent/Legal Guardian Who Had COVID-19 Disease		Children Who Had COVID-19	
	%		%	
21–25	50		16.6	
26–30	23.6		18.4	
31–35	38.3		25.5	
36–40	25.7		16.9	
41–45	33.7		28.8	
46–50	34		26.7	
51–55	26.8		29.2	
56–60	20		30	
61–65	100		100	
66+	33.3		33.3	
Statistical analysis	Mean	0.3854	Mean	0.3254
	Standard Error	0.073407871	Standard Error	0.077210563
	Median	0.335	Median	0.2775
	Standard Deviation	0.232136071	Standard Deviation	0.24416124
	Sample Variance	0.053887156	Sample Variance	0.059614711
	Kurtosis	6.560056789	Kurtosis	8.482739794
	Skewness	2.441174735	Skewness	2.821020663
	Confidence Level (95.0%)	0.166060142	Confidence Level (95.0%)	0.174662429

**Table 4:** Vaccination of adults and children with the mandatory vaccines included in the national scheme.

Age Range for Parents/Legal Guardians	Parents Vaccinated with Vaccines Included in the National Vaccination Schedule		Children Vaccinated with Vaccines Included in the National Vaccination Schedule	
	%		%	
21–25	100		83.3	
26–30	94.7		92.1	
31–35	98.9		97.8	
36–40	97		95.5	
41–45	100		99.3	
46–50	98.9		98.9	
51–55	97.5		97.5	
56–60	100		100	
61–65	100		100	
66+	100		100	
Statistical analysis	Mean	0.987	Mean	0.9644
	Standard Error	0.005663	Standard Error	0.016587
	Median	0.9945	Median	0.9835
	Standard Deviation	0.017907	Standard Deviation	0.052451
	Sample Variance	0.000321	Sample Variance	0.002751
	Kurtosis	1.618865	Kurtosis	4.529729
	Skewness	−1.44253	Skewness	−2.09243
	Confidence Level (95.0%)	0.01281	Confidence Level (95.0%)	0.037522

**Table 5:** Confidence in the mandatory vaccines included in the national scheme and in the optional vaccines (*like rotavirus vaccine, anti hepatitis A and B, meningococcal vaccine, etc.*).

Age Range for Parents/Legal Guardians	Degree of Confidence in Vaccines Included in the National Vaccination Schedule and in Optional Vaccines (Hepatitis B Vaccine, Hexavalent Vaccine, Tuberculosis Vaccine, etc.).			
	Vaccines Included in the National Scheme		Optional Vaccines	
	%		%	
21–25	83.3		83.3	
26–30	81.5		71	
31–35	91.4		72.3	
36–40	95.5		83	
41–45	96.6		81.4	
46–50	93.4		76.9	
51–55	87.8		65.8	
56–60	90		90	
61–65	50		100	
66+	100		83.3	
Statistical analysis	Mean	0.8695	Mean	0.807
	Standard Error	0.044923	Standard Error	0.031258
	Median	0.907	Median	0.822
	Standard Deviation	0.14206	Standard Deviation	0.098848
	Sample Variance	0.020181	Sample Variance	0.009771
	Kurtosis	5.898115	Kurtosis	0.454139
	Skewness	−2.25403	Skewness	0.456268
	Confidence Level (95.0%)	0.101623	Confidence Level (95.0%)	0.070711

**Table 6:** Schedule of anti-COVID-19 vaccination of parents and their children.

Age Range for Parents/Legal Guardians	Vaccination of Parents/Legal Guardians and Children against COVID-19			
	Parents/Guardians		Children	
	%		%	
21–25	41.6		16.6	
26–30	55.2		2.6	
31–35	64.8		6.3	
36–40	79.4		21.3	
41–45	73.5		40.4	
46–50	73.6		58.2	
51–55	58.5		51.2	
56–60	80		70	
61–65	50		50	
66+	83.3		66.6	
Statistical analysis	Mean	0.6599	Mean	0.3832
	Standard Error	0.044901732	Standard Error	0.078654491
	Median	0.6915	Median	0.452
	Standard Deviation	0.141991745	Standard Deviation	0.248727338
	Sample Variance	0.020161656	Sample Variance	0.061865289
	Kurtosis	−1.100961288	Kurtosis	−1.582192121
	Skewness	−0.439693194	Skewness	−0.248886703
	Confidence Level (95.0%)	0.101574775	Confidence Level (95.0%)	0.177928819

**Table 7:** Parents agree to vaccinate their children when they reach the recommended minimum age or when vaccines will be available for their age.

Age Range for Parents/Legal Guardians	Agreement of Parents/Legal Guardians to Vaccinate Children against COVID-19 When They Reach the Minimum Age for Vaccination or When a Vaccine Will Be Available for Their Age	
	%	
21–25	33.3	
26–30	39.4	
31–35	44.6	
36–40	58.8	
41–45	58.9	
46–50	64.8	
51–55	58.5	
56–60	80	
61–65	100	
66+	83.3	
Statistical analysis	Mean	0.6216
	Standard Error	0.065816107
	Median	0.5885
	Standard Deviation	0.208128806
	Sample Variance	0.0433176
	Kurtosis	−0.37428505
	Skewness	0.430758375
	Confidence Level (95.0%)	0.148886379

**Table 8:** The level of readiness of parents vaccinated against COVID-19 to vaccinate their own children.

Age Range for Parents/Legal Guardians	Vaccinating Children against COVID-19—Vaccinated Parents against COVID-19.	
	%	
21–25	15	
26–30	4.7	
31–35	9.8	
36–40	26.8	
41–45	52.2	
46–50	77.6	
51–55	83.3	
56–60	87.5	
61–65	100	
66+	80	
Statistical analysis	Mean	0.04379562
	Standard Error	0.016573612
	Median	0.015815085
	Standard Deviation	0.052410363
	Sample Variance	0.002746846
	Kurtosis	−0.063979027
	Skewness	1.180022504
	Confidence Level (95.0%)	0.037492115

**Table 9:** Readiness of unvaccinated parents against COVID-19 for vaccination of children.

Age Range for Parents/Legal Guardians	Vaccinating Children against COVID-19—Unvaccinated Parents against COVID-19	
	%	
21–25	0%	
26–30	0%	
31–35	0%	
36–40	0%	
41–45	7.5%	
46–50	4.17%	
51–55	5.8%	
56–60	0%	
61–65	0%	
66+	0%	
Statistical analysis	Mean	0.002941176
	Standard Error	0.001807754
	Median	0
	Standard Deviation	0.00571662
	Sample Variance	3.26797E-05
	Kurtosis	5.356401384
	Skewness	2.269834215
	Confidence Level (95.0%)	0.004089423

**Table 10:** Identifying the degree of trust given to “fake news” statements [14].

Fake News Allegations	Disagree		Uncertain		Agree		P
	N	%	N	%	N	%	
There is a global secret society who wants to control the world	370	63.8	80	13.7	131	22.5	P = 0.02
COVID-19 vaccines are made to reduce the population of the Earth (infertility, death etc.)	420	72.2	66	11.3	95	16.3	P > 0.01
Doctors are paid to inoculate children with a vaccine that would help reduce the Earth’s population	441	75.8	68	11.7	72	12.3	P > 0.01
Children who get the COVID-19 vaccine will die in the next few years from inoculated substances	435	74.8	101	17.3	45	7.7	P > 0.01
Children who get the COVID-19 vaccine will become infertile due to inoculated substances	441	75.9	94	16.1	46	7.9	P > 0.01
Vaccination is intended to reduce the number of elderly people	438	75.5	57	9.8	85	14.6	P > 0.01
Vaccination on a global scale aims to enrich vaccine manufacturers	335	57.6	71	12.2	175	30.1	P = 0.03
There is a global secret society that wants to reduce the population of the Earth	405	68.7	58	9.9	118	20.3	P > 0.01
New vaccines based on messenger RNA produce dangerous genetic changes	382	65.7	98	16.8	101	17.3	P < 0.05

## Data Availability

Data can be requested from the corresponding author.
